# Improving Pneumococcal Vaccination Rates in a Community-Based Internal Medicine Resident Clinic

**DOI:** 10.51894/001c.6465

**Published:** 2018-04-27

**Authors:** Cale Sebald, Lana Joubert, Marcella Novakosky, MaryEllen Rosel

**Affiliations:** 1 St. John Oakland-Macomb, Gastrointestinal Medicine Fellow, Madison Heights, MI; 2 Mercy Health Muskegon, Internal Medicine Residency Program, Muskegon, MI

**Keywords:** vaccination protocol, community-based resident clinic, pneumococcal vaccination

## Abstract

**CONTEXT:**

Despite proven benefits of vaccination such as reduced morbidity and mortality, many patients remain out of date on their recommended vaccines. The goal of this pilot project was to develop and test a systematic vaccination review and ordering protocol aimed to increase the percentage of patients who were assessed under current pneumococcal vaccine recommendations by 5%.

**METHODS:**

The study location was set in a community-based internal medicine resident clinic in Muskegon, Michigan, with the patient population coming from the same setting. Data from 50 patients who had completed office visit appointments at a resident clinic from January 2016 through April 7th, 2017 were randomly extracted before implementation of the protocol. Two months post-implementation, the authors obtained office visit data from another randomly selected 50 clinic patients for comparison. The nurses and medical assistants in the office had been educated on the pneumonia vaccine protocol based on CDC (United States Centers for Disease Control and Prevention) vaccination guidelines and state registry records. They were also provided copies of the seven-step vaccine assessment and ordering protocol that included obtaining MCIR (Michigan Care Improvement Registry) data to update the patient’s chart for a possible provider order set. Clinic residents were also educated on CDC pneumonia vaccine guidelines, and the authors posted several guideline related posters on clinic walls.

**RESULTS:**

The authors initially compared the percentage of patients who providers had assessed regarding their vaccination status before protocol implementation to the percentage of patients after protocol implementation. There was a 10% post-implementation increase in pneumonia vaccination assessment.

**CONCLUSIONS:**

Although the results of this pilot project are obviously limited by methodological and sample size characteristics, the initially measured improvements in vaccination status suggests that this type of systematic protocol approach may warrant further testing in similar settings.

## INTRODUCTION

Vaccines are arguably one of the greatest advances in modern medicine and responsible for the prevention, and in some cases, the almost complete eradication of certain diseases.^1^ In addition, vaccinations have been proven to reduce both morbidity and mortality.^2^ However, despite vaccination benefits, millions of adult patients remain behind on their vaccinations as outlined by The United States Centers for Disease Control and Prevention (CDC).^3^

In 1942, the CDC, a federal agency, was established to fight the spread of malaria in the southern United States.^4^ Since that time, the organization has expanded its focus to include general public health efforts to prevent and control a number of hazardous infectious and chronic diseases. The CDC continues to conduct research and implement disease prevention strategies that include formulation of national vaccination recommendations.^4^

The large number of unvaccinated persons has a significant impact on the health and economics of individuals and populations alike. Specifically, there has been a more than 92% reduction in incidence and a 99% decline in deaths attributable to numerous vaccine-preventable illnesses from 1980 to the present compared to pre-vaccine eras.^5^

To improve vaccination rates and break down barriers to immunization, several states have developed their own databases for tracking completed immunizations. In 1996, the Michigan Childhood Immunization Registry was developed by public health officials to track childhood immunizations. In 2006, the registry was expanded to include adult immunizations and renamed the Michigan Care Improvement Registry (MCIR).^6^ However, the registry continues to depend on clinic staff consistently reporting when an immunization is administered. Therefore, data in the MCIR the database is not always complete and accurate.

Since the implementation of statewide vaccination databases, there have also been several ongoing barriers identified to adult vaccinations. One study found that false assumptions, such as, “healthy people don’t need vaccinations,” influenced some patients deciding to not agree to vaccinations.^7^ This same research group also discovered that another important practice barrier was that many physicians were simply prioritizing other patient issues.^7^ Additionally, provider concerns about vaccine efficacy and adverse events following vaccine administration have also been cited in a separate study.^8^ A sample of internal medicine residents in another project indicated that less than half of sample respondents know what CDC recommendations for pneumonia boosters are and whether revaccination guidelines existed.^9^

### History of Pneumococcal Vaccine

The development of a Streptococcus pneumoniae vaccination started as early as 1911. These developmental efforts slowed down significantly after the discovery of penicillin. Patients continued to die from this infection despite penicillin treatments, and efforts were resumed to formulate an effective pneumococcal vaccine. In 1977, the first pneumococcal polysaccharide vaccine was licensed. This vaccine contained the polysaccharide (carbohydrate coating of the Streptococcal bacteria) antigens from 14 different sorts of pneumococcal bacteria. In 1983, the PPSV 23 (pneumococcal polysaccharide vaccine 23) which contained 23 types of pneumococcal polysaccharide antigens, replaced this vaccine.^10^

In 2000, the first pneumococcal conjugate vaccine was licensed covering seven different types of pneumococcal bacteria.^11^ This conjugate vaccine was different from the PPSV 23 in that the pneumococcal antigen is conjugated (i.e., attached) to a diphtheria toxin, another bacterium that the body has a much stronger immunologic response to. Combining these two bacterial products allows the body to develop greater immunity against the pneumococcal bacteria by associating it with the diphtheria toxin, than it would develop if only exposed to the pneumococcal bacteria.^11^

The efficacy of the PCV13 vaccine was determined in the CAPiTA trial (Community Acquired Pneumonia Immunization Trial in Adults).^11^ The study showed 46% efficacy against vaccine-type pneumococcal pneumonia, 45% efficacy against vaccine-type non-bacteremic pneumococcal pneumonia, and 75% efficacy against vaccine-type invasive pneumococcal disease.^11^ The efficacy of the PPSV23 vaccine was evaluated in a meta-analysis in 2013. This study determined that PPSV 23 significantly reduced the risk of invasive pneumococcal disease.^11^

### Purpose of Study

The goal of this quality improvement (QI) pilot project was to develop and test a systematic vaccination review and ordering protocol aimed to increase the percentage of patients who had been assessed under current pneumococcal vaccine recommendations by 5%. The population of resident-assigned patients in the project clinic tended to be quite unhealthy, with about 3,400 annual patient encounters and 700 missed office visits.

## METHODS

An information technologist familiar with the Athena electronic health record (EHR) used in the authors’ resident clinic was contacted to extract data concerning all patient encounters from January 2016 until April 7^th^, 2017. They used Microsoft Excel software to house data from 50 random patient encounters out of several thousand patient encounters during that period. Random numbers were then assigned to each encounter. The authors then selected the “sort” option to sort the random numbers from smallest to largest values. They then selected the first 50 numbered patients for review.

To obtain an initial estimate of the overall baseline vaccination assessment rate in the resident clinic, the authors randomly selected a sample of 50 random patient encounters and reviews of their pneumonia vaccination status was determined. Then, after implementing an internally developed pneumococcal vaccination assessment and ordering protocol, this vaccination assessment rate was again measured from another random sample.

The authors examined data in the clinic EHR and MCIR registry and cross-referenced records to determine whether each sample patient had been assessed regarding their pneumococcal vaccine status observing the CDC guidelines depicted in Appendix 1. Since, both the EHR and MCIR were dependent on clinic staff reporting administered vaccinations, these databases who were not entirely accurate and may have not differentiated between the PCV13 and PPSV 23. If the patient appeared to meet the indication for receiving a pneumococcal vaccine but had received an alternate vaccine (either PCV13 or PPSV23), then the patient was still concluded to have had their vaccination status reviewed.

On April 10, 2017, authors implemented their pneumococcal vaccine review and ordering protocol. The nurses and medical assistants (MAs) in the office were educated concerning CDC pneumonia vaccine guidelines and asked to follow the seven-step internally developed clinic protocol:

1. Check to see if a MCIR report had been logged in for the patient; if not, proceed to Step 2. If a report had been logged, inform the provider and continue with your regular intake procedures.
2. Obtain the MCIR data for each patient and update their EHR to reflect previously-administered vaccines.
3. If the patient had not received or been assessed for a pneumococcal vaccine, the patient was asked if they would like to receive a vaccination that day.
4. If the patient wished to receive a vaccine, create a pneumococcal vaccine order in patient’s encounter record and administer the pneumococcal vaccine.
5. Document in patient’s EHR refusal reason if patient did not wish to be vaccinated.
6. Notify provider of patient’s vaccination status if order had been created, if vaccine was given, and whether the patient had refused.
7. Document in chart that MCIR had been reviewed, and the date during which it was reviewed.

Clinic residents were also educated concerning CDC pneumonia vaccine guidelines, and the authors posted several posters containing these guidelines for pneumonia vaccination on the clinic walls.

Two months after protocol implementation, data were extracted concerning all patient clinic encounters since January 2016. Using the same random selection sequence, fifty new discrete patients were randomly chosen from the 400-500 documented encounters during this period. Again, the authors used the Athena EHR and MCIR to determine if each post-implementation patient had been reviewed for a possible pneumococcal vaccine.

## RESULTS

In the initial pre-intervention group of 50 patients, 23 (46%) patients had not had their pneumonia vaccination status assessed, and 27 (54%) had. In the second post-intervention group of 50 patients after implementation of the protocol, 18 (36%) patients had not had their pneumonia vaccination status assessed and 32 (64%) had.

Based on our comparative random sample subgroups, we may have achieved a 10% overall improvement in rates of pneumonia vaccination status assessment after clinic protocol implementation. We should acknowledge that two months may not have really been enough time to observe a significant or sustainable pre-to-post implementation result. Unfortunately, evaluating MCIRs and EHR records for more than 50 patients pre- and 50 post-implementation patients would have required considerably more resources.

Please note that patients who did not require vaccination because they were under age 65 or had no qualifying comorbidities were treated as having had their pneumonia vaccination status reviewed. For this pilot project, those type of patients were treated in this way due to an assumption that their provider had determined that they did not require a pneumococcal vaccine at the time. However, if we had not chosen to include these patients in our calculations, the final outcome may be different and is definitely something that could be studied in the future. Certainly, our actual percentage of improvement in pneumococcal vaccination assessment could have been considerably higher or lower.

Furthermore, as this was a QI pilot project, (i.e., not a true research study), the socio-demographic characteristics of the pre and post-implementation subgroups were not inferentially examined due to the relatively small sample size. In larger future studies, researchers will need to compare pre and post-protocol implementation rates to help ensure relatively equivalency of comparison subgroups.

We did not revise the project protocol at all during our two months after implementation. The workflow between the clinical staff and the residents first appeared to go smoothly and accomplish what we hoped the protocol would achieve. Over time, however, we did note that the protocol was not followed for every patient across residents and MAs. It also seemed to become easier to disregard this protocol when the clinic was busy and understaffed during the post-implementation period. This phenomenon is important to consider since we did not systematically monitor when the protocol was being followed or not. In hindsight, we should have formulated a way to improve consistent protocol adherence for every patient encounter. This could constitute an entirely new QI project.

## DISCUSSION

As discussed earlier, there are many barriers to completion of primary care pneumococcal vaccination. Patient vaccinations remain incredibly important due to their ability to lower mortality and morbidity and healthcare expenditures.^2^ During the implementation of our vaccination assessment protocol, we also encountered some unexpected barriers from elements of our own healthcare system. Shortly after implementation of our protocol, an email came from hospital administration to providers regarding pneumonia vaccinations. This email stated that our healthcare system would not cover pneumococcal vaccinations for patients with certain types of inadequate insurance coverage. Instead, residents were directed to print a prescription and have the patient obtain the vaccine elsewhere.

Regardless of our project limitations, it is notable that we were able to increase pneumonia vaccination assessment rates by approximately 10% in just two months. Our protocol rollout may have helped address provider lack of awareness regarding vaccination eligibility guidelines. By educating the physicians, nurses, MAs, and posting CDC guideline posters in visible clinic locations, we hoped to ensure that healthcare staff remembered to assess patients’ pneumonia vaccination status.

In 2004, a randomized trial study examined whether standing EHR physician orders or physician EHR prompts concerning pneumococcal immunizations were more effective. This study group found that automatically generated physician orders were much more effective in increasing pneumococcal vaccination rates than EHR reminders to order a pneumococcal vaccination.^12^

Our study was somewhat similar to this 2004 trial in that we were essentially creating a protocol to remind physicians to vaccinate when indicated. As noted earlier, this protocol was not consistently followed for every patient encounter due to provider oversight and competing patient care demands. Additionally, post-project initiation of this protocol by the MAs and physicians in the resident clinic has seemed to decline significantly. Similar to this 2004 study, we have since concluded that an EHR-generated order set for the pneumococcal vaccination might have been more successful to prompt provider assessment of patients’ pneumococcal vaccination status.

### Protocol Revisions

Although our project very likely lacked an adequate level of statistical power to conduct inferential statistical procedures, we did measure a small improvement in pneumococcal vaccination review rates. In the future, we are hoping to increase pneumococcal vaccination rates further in our clinic by creating a pre-completed EHR order set that will include orders for both the PCV 13 and PPSV 23 vaccinations. The MAs will be responsible for selecting this order set every time they room any incoming patient. This would require the physician to assess each patient’s pneumococcal vaccination status at every encounter. At the end of the encounter, the physician would then delete the order set if pneumococcal vaccination was not indicated or leave the recommended vaccination in the order set for the patient to receive the vaccination.

## CONCLUSIONS

Based on these initial results, our pneumococcal vaccination protocol may have helped facilitate improved adherence to the national CDC guidelines. Unfortunately, it appeared to be difficult to maintain staff and physician compliance with our protocol. Future larger studies testing the implementation of more streamlined vaccination order sets to address barriers to pneumococcal vaccination may increase adherence further than vaccination review protocols.

### Conflict of Interest

The authors declare no conflict of interest.

## APPENDIX 1

### CDC Pneumococcal Guidelines

1. Adults age 19 through 64 with the following comorbidities should receive PPSV23:
  - CHF and cardiomyopathies (excluding HTN)
  - Chronic lung disease including COPD, emphysema, and asthma
  - Chronic liver disease including cirrhosis, alcoholism, or DM
  - Cigarette smokers
  - At age 65 or older, they should receive PCV13 and another dose of PPSV23 at least 1 year after PCV13 and at least 5 years after the most recent dose of PPSV23
2. Adults age 19 or older with immunocompromising conditions or anatomical/ functional asplenia should receive PCV13 and PPSV23 at least 8 weeks after PCV13 followed by a second dose of PPSV23 at least 5 years after the first PPSV23. Immunocompromising conditions and asplenia are defined as follows:
  - Congenital or acquired immunodeficiency including B or T lymphocyte deficiency, complement deficiencies, and phagocytic disorders (This excludes chronic granulomatous disease)
  - HIV, chronic renal failure and nephrotic syndrome, leukemia, lymphoma,
  - Hodgkin disease, generalized malignancy, and multiple myeloma, solid organ transplant, and iatrogenic immunosuppression including long term systemic corticosteroid and radiation therapy
  - Sickle cell disease and other hemoglobinopathies, congenital or acquired asplenia, splenic dysfunction, and splenectomy
3. Adults aged 19 years or older with CSF leak or cochlear implant should receive PCV13 followed by PPSV23 at least 8 weeks after PCV13.
  - If the most recent dose of PPSV23 was administered before age 65, at age 65 or older administer another dose of PPSV23 at least 8 weeks after PCV13 and at least 5 years after the most recent dose of PPSV23
4. Adults aged 65 years or older should receive PCV13 followed by PPSV23 at least one year after PCV13
  - When both PCV13 and PPSV23 are indicated, PCV13 should be administered first
  - If PPSV23 has previously been administered, PCV13 should be administered at least 1 year after PPSV23
